# Comparison of Mouse and Human Retinal Pigment Epithelium Gene Expression Profiles: Potential Implications for Age-Related Macular Degeneration

**DOI:** 10.1371/journal.pone.0141597

**Published:** 2015-10-30

**Authors:** Anna Bennis, Theo G. M. F. Gorgels, Jacoline B. ten Brink, Peter J. van der Spek, Koen Bossers, Vivi M. Heine, Arthur A. Bergen

**Affiliations:** 1 Department of Clinical Genetics, Academic Medical Centre, Amsterdam, The Netherlands; 2 The Netherlands Institute for Neuroscience (NIN-KNAW), Royal Netherlands Academy of Arts and Sciences, Amsterdam, The Netherlands; 3 University Eye Clinic Maastricht, Maastricht University Medical Centre+, Maastricht, The Netherlands; 4 Department of Bioinformatics, Erasmus University Medical Center, Rotterdam, The Netherlands; 5 Laboratory for Neuroregeneration, the Netherlands Institute for Neuroscience, Royal Netherlands Academy of Arts and Sciences, Amsterdam, The Netherlands; 6 Department of Pediatrics / Child Neurology, Neuroscience Campus Amsterdam, VU University Medical Centre, Amsterdam, The Netherlands; 7 Department of Complex Trait Genetics, Center for Neurogenomics and Cognitive Research, Neuroscience Campus Amsterdam, VU University, Amsterdam, The Netherlands; 8 Department of Ophthalmology, Academic Medical Centre, Amsterdam, The Netherlands; University of Cologne, GERMANY

## Abstract

**Background:**

The human retinal pigment epithelium (RPE) plays an important role in the pathogenesis of age related macular degeneration (AMD). AMD is the leading cause of blindness worldwide. There is currently no effective treatment available. Preclinical studies in AMD mouse models are essential to develop new therapeutics. This requires further in-depth knowledge of the similarities and differences between mouse and human RPE.

**Methods:**

We performed a microarray study to identify and functionally annotate RPE specific gene expression in mouse and human RPE. We used a meticulous method to determine C57BL/6J mouse RPE signature genes, correcting for possible RNA contamination from its adjacent layers: the choroid and the photoreceptors. We compared the signature genes, gene expression profiles and functional annotations of the mouse and human RPE.

**Results:**

We defined sets of mouse (64), human (171) and mouse–human interspecies (22) RPE signature genes. Not unexpectedly, our gene expression analysis and comparative functional annotation suggested that, in general, the mouse and human RPE are very similar. For example, we found similarities for general features, like “organ development” and “disorders related to neurological tissue”. However, detailed analysis of the molecular pathways and networks associated with RPE functions, suggested also multiple species-specific differences, some of which may be relevant for the development of AMD. For example, CFHR1, most likely the main complement regulator in AMD pathogenesis was highly expressed in human RPE, but almost absent in mouse RPE. Furthermore, functions assigned to mouse and human RPE expression profiles indicate (patho-) biological differences related to AMD, such as oxidative stress, Bruch’s membrane, immune-regulation and outer blood retina barrier.

**Conclusion:**

These differences may be important for the development of new therapeutic strategies and translational studies in age-related macular degeneration.

## Introduction

Age related macular degeneration (AMD) is the leading cause of blindness worldwide. The disease affects 4% of the population over age 60. With the increase of the aging population, AMD is becoming an even more important public health issue. The etiology of AMD remains largely unknown. The first clinical manifestations of the disease include the appearance of sub-retinal drusen and pigmentary or degenerative changes of the RPE. Ultimately, the disease affects the RPE, Bruch’s membrane (BM), photoreceptors (PR) and choriocapillaries (CH). We focused this study on the RPE.

The RPE is a monolayer of pigmented neuro-epithelial cells, which forms part of the outer blood-retina barrier. It closely interacts with the PR to maintain visual function. The apical membrane of the RPE faces the photoreceptor outer segments and its basolateral membrane faces the BM. The BM separates the RPE from CH, which nourishes the RPE and outer layers of the retina [1]. In healthy eyes, BM functions as a structural support that is permeable to fluid and small molecules. Additionally it acts as a physical barrier, containing anti-angiogenic molecules, which protect the retina against neovascularization [2,3].

A healthy RPE is essential for visual function. It supplies the PR with nutrients, absorbs the excess light energy focused by the lens on the retina, recycles retinal from the PR, regulates the ion balance in the sub retinal space and maintains the function and survival of the PR by phagocytosis of the shed photoreceptor outer segments [1]. Failure of any of these functions can lead to degeneration of the retina, loss of visual function and, eventually, blindness in retinal diseases such as AMD or retinitis pigmentosa.

In AMD, RPE dysfunction or degeneration leads to a dystrophy of the PR and thereby vision loss [4]. The early stage of AMD is characterized by the presence of drusen and vision loss is relatively mild. Later stages of the disease involve two forms: the dry form (geographic atrophy) and the wet form (choroidal neovascularization). Both forms affect about half of the late stage AMD patients. AMD has a multifactorial etiology [5], and is caused by a variety of environmental and genetic risk factors [4]. There is evidence that positive life style changes (quit smoking; healthy food) and dietary supplements (Zn^2+^) may postpone the onset or progression of the disease [6]. Patient-unfriendly, repeated intra-ocular injections with anti-VEGF may temporarily halt the progression of the wet form of AMD. However, it does not prevent the atrophy of RPE and PR [7,8]. Once vision is lost, a possible (future) cure for AMD may be cell replacement therapy. Pre-clinical experiments indicate that transplantation of stem cell derived RPE cells can successfully be used to rescue PR and vision [9–11]. However, these preclinical studies are predominantly performed in mice. To translate results and start clinical studies in man further knowledge of the similarities and differences between mouse and human RPE is essential.

In this study we compared the gene expression profiles and functional annotation of mouse and human RPE on a single microarray platform to further improve translational studies.

## Results

**Fig 1:**
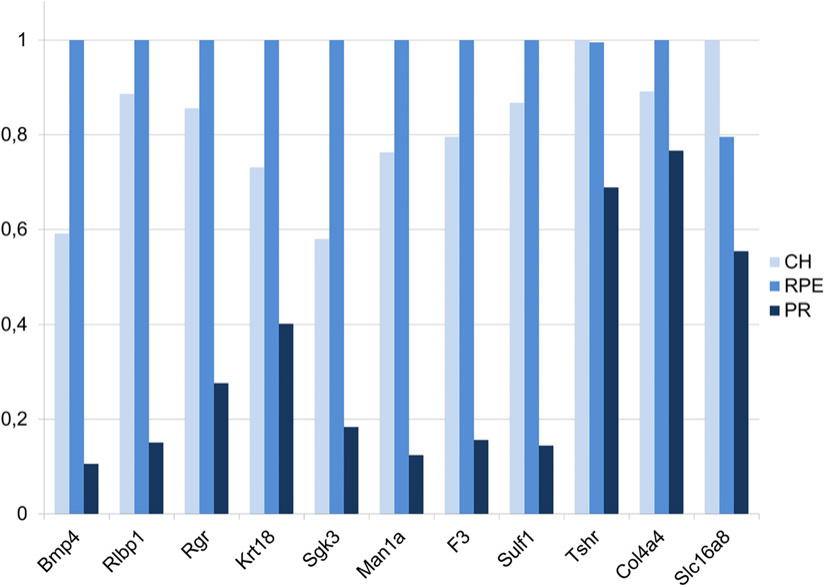
Confirmation of microarray results by sqRT-PCR. Beta-actin (*Bact*), a housekeeping gene, was used to normalize gene expression in mouse CH, RPE and PR. The light blue bars indicate expression levels in CH, the blue bars expression levels in the RPE and the dark blue bars indicate expression levels in PR. Similar to the microarray data the expression level is highest in the RPE and lowest in the PR. The sqRT-PCR results confirm our findings; however *Tshr* and *Slc16a8* show expression lower in RPE compared to choroid. Overall, the sqRT-PCR confirmation rate in this, and in all our previous studies (combined), using exactly the same methodology and platform to investigate neuroepithelia from human donor eyes and brains was 87% [12–14].

First, we determined the gene expression profiles of the mouse RPE, CH and PR (raw data available in the Gene Expression Omnibus database with the accession number GSE66916). We confirmed our microarray methodology by checking the expression of (well established) RPE genes using semi-quantitative RT-PCR (sqRT-PCR) (Fig 1 and S1 Fig). Subsequently, we determined mouse and human RPE signature genes, we defined the functionalities of the gene expression profiles of mouse and man, and we analyzed the most extreme differences in RPE gene expression between the two species. Also these results were partly confirmed using sqRT-PCR (S2 Fig).

### Mouse, human and inter-species RPE signature genes

**Fig 2:**
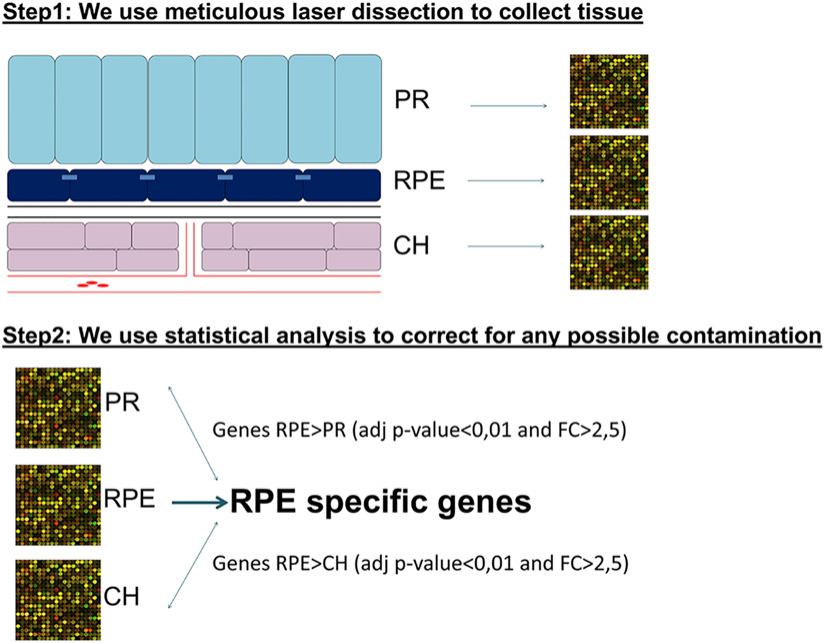
Strategy to select RPE signature genes. In the first step of this strategy we laser dissect the RPE (and its adjacent layers, the CH and PR) for specific tissue collection. In the second step we statistically correct for possible contamination by adjacent layers.

In our lab, we previously designed a new strategy to select RPE signature genes (Fig 2). RPE specificity was determined by comparison of the gene expression levels between the RPE and its adjacent layers: the CH and PR [12]. In the current study, we applied this strategy to the mouse retina in order to select mouse RPE signature genes.

We selected the genes that have a significant higher expression level in the mouse RPE compared to their expression in the CH and the PR, with a fold change (FC) higher than 2.5 and a B-H adjusted p-value<0.01. This resulted in a list of 64 genes that are specifically expressed in the RPE relative to both its adjacent layers; the CH and PR. We annotated this set the “Mouse RPE signature genes” dataset (see Table 1).

**Table 1:** Our “Mouse RPE signature genes” dataset: 64 mouse RPE genes with an average expression of at least 2.5 fold higher in the mouse RPE than in both the PR and the CH with an adjusted p-value smaller than 0.01.

CH<RPE>PR genes		RPE compared to CH		RPE compared to PR	
GeneName	SystematicName	adj.P.Val	FC RPE-CH	adj P value	FC RPE-PR
Rgr	ENSMUST00000022338	5,93E-03	4,9	5,90E-06	306,1
LOC100045988	XM_001475309	6,03E-03	4,6	3,81E-03	3,5
Pon1	NM_011134	1,01E-03	4,2	2,10E-07	95,8
Rdh10	NM_133832	1,46E-03	4,1	9,38E-08	75,1
Arl6ip1	NM_019419	4,41E-03	3,2	2,73E-06	24,6
Rlbp1	NM_020599	5,78E-03	3,2	4,29E-07	42,4
Tbx5	NM_011537	2,65E-03	3,2	3,61E-04	3,5
Bmp4	NM_007554	3,03E-03	3,2	2,31E-05	47,9
F3	NM_010171	1,42E-03	3,1	4,09E-07	102,2
5730469M10Rik	NM_027464	2,28E-03	3,1	1,03E-06	14,0
Rrh	NM_009102	2,40E-03	3,1	8,94E-08	47,2
Man1a	NM_008548	2,07E-04	3,0	6,11E-08	10,3
Sema3c	NM_013657	5,52E-04	2,9	9,66E-08	504,3
Vldlr	NM_013703	1,35E-03	2,9	1,79E-05	4,6
Atp1b1	NM_009721	2,54E-03	2,9	1,29E-07	34,1
Ctsd	NM_009983	6,72E-03	2,9	6,81E-05	5,7
Cspg5	NM_001166273	5,34E-03	2,9	1,94E-06	15,1
Cldn2	NM_016675	6,79E-04	2,9	6,75E-07	7,8
Sulf1	NM_172294	4,05E-04	2,9	2,38E-07	22,9
BC048943	NM_001127685	1,48E-03	2,9	8,23E-05	3,4
Slc39a12	NM_001012305	6,97E-04	2,9	8,03E-08	123,3
Loxl4	NM_001164311	4,47E-04	2,8	7,52E-07	13,2
NAP114398-1	NAP114398-1	5,88E-04	2,8	2,70E-07	9,4
Slc1a1	NM_009199	6,31E-03	2,8	1,30E-07	27,2
Slc6a13	NM_144512	3,86E-03	2,8	9,05E-08	49,1
Car12	NM_178396	5,92E-03	2,8	2,86E-07	34,5
Iqgap2	NM_027711	3,55E-04	2,8	5,11E-08	13,4
Hist2h2aa1	NM_013549	2,53E-04	2,8	2,57E-07	5,7
Tgfa	NM_031199	1,07E-03	2,8	2,66E-07	11,9
Spon1	NM_145584	3,68E-04	2,7	2,54E-07	7,4
Flot2	NM_008028	4,72E-03	2,7	1,50E-05	7,1
Tmem27	NM_020626	1,64E-03	2,7	3,15E-05	108,8
Trhde	NM_146241	1,06E-03	2,7	7,15E-08	19,8
Hist2h4	NM_033596	8,85E-03	2,7	5,29E-05	7,0
Itgb8	NM_177290	2,57E-03	2,7	4,33E-07	14,9
Cbfa2t3	NM_001109873	6,18E-03	2,7	2,75E-06	11,8
Tcfl5	NM_178254	1,92E-03	2,7	4,74E-07	12,2
Adora2b	NM_007413	3,61E-04	2,7	1,30E-07	37,0
Spock1	NM_009262	2,29E-03	2,7	1,28E-06	9,3
Gpam	ENSMUST00000086868	8,06E-03	2,7	1,09E-03	3,0
Acsl6	NM_001033599	2,80E-03	2,7	2,96E-04	2,8
Lrp2	NM_001081088	6,73E-03	2,7	3,74E-06	10,7
Slc6a20a	NM_139142	5,91E-04	2,6	3,38E-07	7,6
Nt5dc2	NM_027289	1,30E-03	2,6	9,65E-07	7,7
Krt18	NM_010664	2,04E-03	2,6	1,73E-06	7,2
Slc16a8	NM_020516	1,87E-03	2,6	2,46E-07	14,9
Gabrb3	NM_008071	3,18E-04	2,6	4,83E-06	3,3
Mogat1	NM_026713	2,79E-03	2,6	5,42E-07	12,9
Hkdc1	NM_145419	2,81E-03	2,6	7,31E-06	5,9
Tmem56	NM_178936	4,12E-03	2,6	1,71E-08	148,2
Col4a4	NM_007735	2,96E-03	2,6	3,44E-06	4,0
Pebp4	NM_028560	4,39E-04	2,6	4,15E-08	13,5
Trpm3	NM_001035246	1,61E-04	2,6	5,35E-09	23,2
Hist1h4i	NM_175656	1,08E-03	2,6	4,09E-06	4,7
A2m	NM_175628	1,55E-03	2,5	8,00E-05	3,0
Bphl	NM_026512	8,30E-04	2,5	2,48E-06	3,1
Slc7a10	NM_017394	1,64E-03	2,5	3,27E-08	33,6
Tshr	NM_011648	4,22E-04	2,5	1,22E-08	58,3
Car14	NM_011797	3,58E-03	2,5	2,60E-07	18,4
Adra2c	NM_007418	4,16E-03	2,5	4,30E-08	54,8
Fam13a	NM_153574	5,08E-04	2,5	2,20E-08	19,6
Sgk3	NM_133220	3,38E-03	2,5	3,87E-06	7,0
Pde4b	NM_019840	5,03E-04	2,5	8,98E-07	5,0
Slco1a4	NM_030687	3,07E-04	2,5	6,65E-09	32,7

Using the same cut-off criteria; we determined a set of genes that is specific for the mouse CH compared to the RPE and a set of genes mouse PR specific when compared to the RPE (S1 Table).

**Fig 3:**
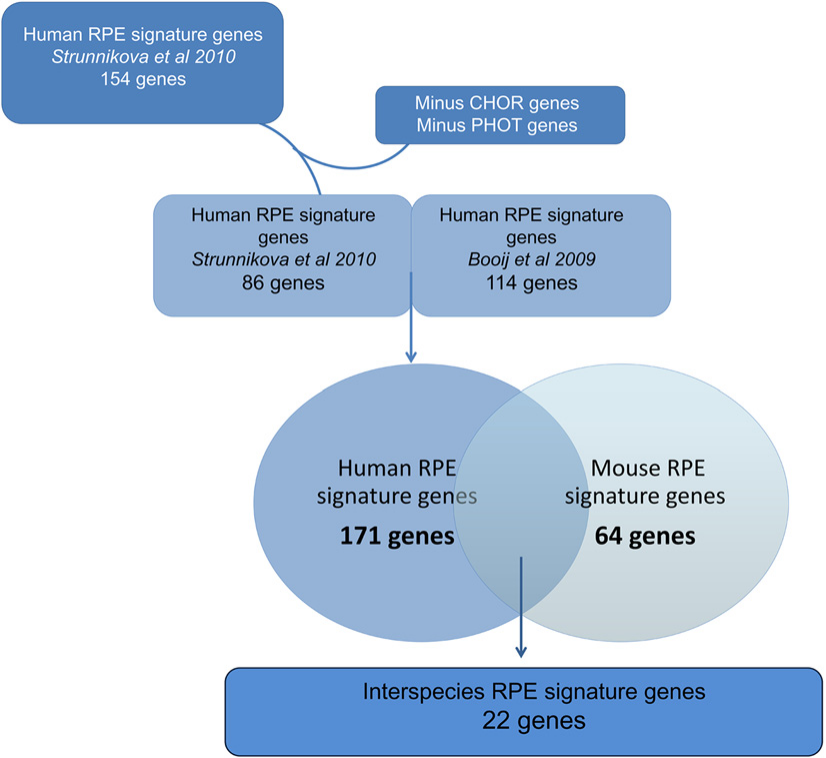
Strategy to determine “Interspecies RPE signature genes”. Schematic overview of our comparison strategy: our “Mouse RPE signature genes” dataset and “Human RPE signature genes” dataset, which contains (a modification of) two human RPE transcriptome datasets [12,15]. This resulted in a new dataset, “Interspecies RPE signature genes”.

We next defined a new “Human RPE signature genes” dataset. We carefully selected two previously published human RPE specific gene expression datasets for a comprehensive comparison between mouse and human RPE (Fig 3) [12,15]. The first study was conducted in our lab using a similar methodology for determining RPE specific gene expression resulting in identification of 114 RPE specifically expressed genes [12]. The second microarray study included multiple RPE types but the investigators did not correct for possible contamination of adjacent tissues [15]. For the latter dataset, we removed possible CH and PR RNA contamination (see Methods), and generated a list of 86 human RPE specifically expressed genes. We subsequently merged the two human RPE specific gene expression datasets, resulting in 171 human RPE signature genes (S2 Table).

Finally, in order to facilitate comparative retinal studies between mouse and human, we aimed to develop a list of interspecies RPE signature genes. We determined the overlap between the mouse (64) and the human (171) RPE signature gene lists, resulting in an interspecies RPE signature gene list of 22 genes (Table 2).

**Table 2:** The 22 signature genes that are specifically expressed in both RPE in mouse and in human.

Gene Symbol	Genbank ID Mus Musculus	Genbank Homo Sapiens
**ADORA2B**	**NM_007413**	**NM_000676**
G-protein coupled adenosine receptor. This integral membrane protein stimulates adenylate cyclase activity in the presence of adenosine.		
**BMP4**	**NM_007554**	**NM_001202**
A member of the bone morphogenic protein family which is part of the transforming growth factor-beta superfamily. The superfamily includes large families of growth and differentiation factors.		
**CA14**	**NM_011797**	**NM_012113**
Carbonic anhydrases are a large family of zinc metalloenzymes that catalyze the reversible hydratyion of carbon dioxide.		
**CSPG5**	**NM_001166273**	**NM_001206942.1 **
A proteoglycan that may function as a neural growth and differentiation factor.		
**CTSD**	**NM_009983**	**NM_001909.4**
An aspartic protease resident in endosomal and lysosomal compartments of all eukaryotic cells.		
**GPAM**	**ENSMUST00000086868**	** **
A mitochondrial enzyme which prefers saturated fatty acids as its substrate for the synthesis of glycerolipids.		
**ITGB8**	**NM_177290**	**NM_002214.2**
Cell surface adhesion receptor mediating cell-adhesion to extra cellular matrix or to other cells, through hetero dimerization and connecting to the cytoskeleton and various signaling molecules within cells.		
**KRT18**	**NM_010664**	**NM_000224**
Keratin 18 is a type I cytokeratin (this type constitutes the type I intermediate filaments of the intracytoplasmatic cytoskeleton, which is present in all mammalian epithelial cells), together with Krt8 is the most common found product of the intermediate filament gene family. They are expressed in single layer epithelial tissues of the body.		
**RDH10**	**NM_133832**	**[NM_172037**
A retinol dehydrogenase, which converts all-trans-retinol to all-trans-retinal, with preference for NADP as a cofactor.		
**RGR**	**ENSMUST00000022338**	**NM_002921**
A putative retinal G-protein coupled receptor and acts as a photoisomerase to catalyze the conversion of all-trans-retinal to 11-cis-retinal.		
**RLBP1**	**NM_020599**	**NM_000326**
Retinaldehyde binding protein 1. carries 11-cis-retinaldehyde or 11-cis-retinal as physiological ligands. It may be a functional component of the visual cycle.		
**SEMA3C**	**NM_013657**	**NM_006379.3**
Binds to plexin family members and plays an important role in the regulation of developmental processes.		
**SLC16A8 [MCT3]**	**NM_020516**	**NM_013356**
Belongs to a family of monocarboxylate transporters. It is expressed in the basolateral membrane of the RPE.		
**SLC39A12**	**NM_001012305**	**NM_152725**
Zinc transporter, which is a cofactor for hundreds of enzymes and therefore normal cell function.		
**SLC6A13**	**NM_144512**	**NM_016615**
Encodes a sodium- and chloride-dependent GABA transporter [GAT2]		
**SLC6A20**	**NM_139142**	**NM_020208**
Encodes an amino acid transmembrane transporter that mediates the transport of small hydrophilic substances across cell membranes.		
**SLC7A10**	**NM_017394**	**NM_019849**
Encodes an amino acid transmembrane transporter that mediates high-affinity transport of D-serine and several other neutral amino acids.		
**SPOCK1**	**NM_009262**	**NM_004598**
Encodes he protein core of a seminal plasma proteoglycan containing chondroitin- and heparin-sulfate chains.		
**SULF1**	**NM_172294**	**NM_015170**
Enzyme which can modulate the activity if heparan sulfate, thereby influencing the regulation of cell growth, proliferation, differentiation and migration.		
**TMEM27**	**NM_020626**	**NM_020665**
binds to amino acid transporters and regulates their expression on the plasma membrane		
**TMEM56**	**NM_178936**	**NM_152487**
function unknown		
**TRPM3**	**NM_001035246**	**NM_206948**
Belongs to the family of transient receptor potential channels. TRP channels are cation-selective channels important for cellular calcium signaling and homeostasis.		

Derived from a comparison between our “Mouse RPE signature genes” dataset (this study) and two (modified) studies on the human RPE transcriptome [12,15]. We show the gene symbol, genbank ID for both species and the GO annotation of each gene.

### Gene expression profiles and functions of the mouse and human RPE

Using our previously published methodology [12–14], we analyzed the highest expressed genes (highest 10^th^ percentile: >90^th^P) of our mouse gene expression dataset (designated “Mouse high RPE gene expression”) to determine the most important functionalities of the mouse RPE. Subsequently, we compared the gene expression pathways and functional annotations of the mouse and human RPE. The latter dataset was available from our previous studies (“Human high RPE specific gene expression dataset” [12]. We used the Ingenuity Knowledge Database to determine biological functions, canonical pathways and molecular networks specific for mouse and human RPE *in vivo*.

**Fig 4:**
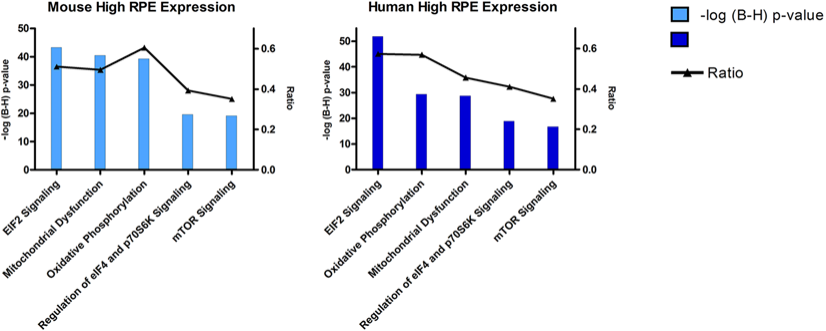
Most significant canonical pathways identified by Ingenuity for the “Mouse High RPE gene expression” and “Human High RPE expression gene expression” datasets. The left y-axis displays the–log of Benjamini-Hochberg corrected p-value. The right y-axis displays the ratio of the number of genes derived from our dataset, divided by the total number of genes in the pathway. The blue line indicates the threshold of the BH corrected p-value of 0.1.

Functional annotation yielded statistically significant **biological functions** that were the same for mouse and human (Table 3). We also found that many important **canonical pathways** for mouse and human RPE were similar. A summary of these findings is presented in Fig 4.

**Table 3:** Overview of the major biological functions found in a functional annotation by Ingenuity of the “Mouse High RPE gene expression” and “Human High RPE gene expression” datasets.

Mouse High RPE expression		Human High RPE expression	
Disease and Disorders	p-value	Disease and Disorders	p-value
Neurological Disease	7,31E-51-9,20E-05	Neurological Disease	7,54E-57-2,18E-05
Psychological Disorders	8,09E-44-9,20E-05	Psychological Disorders	2,04E-49-2,26E-07
Skeletal and Muscular Disorders	2,84E-41-2,62E-05	Skeletal and Muscular Disorders	5,84E-47-2,18E-05
Infectious Disease	5,89E-36-9,33E-05	Hereditary Disorder	1,77E-39-2,18E-05
Hereditary Disorder	6,10E-34-4,77E-05	Infectious Disease	2,29E-32-1,08E-05
**Molecular and Cellular Functions**	** **	**Molecular and Cellular Functions**	
Cellular Growth and Proliferation	7,20E-39-8,57E-05	Cell Death and Survival	3,55E-42-2,06E-05
Cell Death and Survival	3,43E-38-5,57E-05	Cellular Growth and Proliferation	6,25E-37-1,64E-05
Cell Morphology	5,70E-25-9,33E-05	Protein Synthesis	1,77E-22-2,14E-05
Protein Synthesis	5,77E-22-9,28E-06	Cell Morphology	9,15E-21-1,66E-05
Cellular Development	3,25E-17-8,57E-05	Gene Expression	2,01E-20-2,15E-05
**Physiological System Development & Function**	** **	**Physiological System Development & Function**	** **
Organismal Survival	7,97E-23-7,93E-06	Organismal Survival	3,18E-23-3,18E-23
Embryonic Development	9,01E-18-8,15E-05	Organismal Development	2,63E-16-2,01E-05
Organ Development	9,01E-18-8,15E-05	Nervous System Development and Function	3,17E-16-1,66E-05
Organ Morphology	9,01E-18-9,33E-05	Embryonic Development	2,01E-14-1,65E-05
Organismal Development	9,01E-18-8,15E-05	Organ Development	3,73E-13-1,45E-05

The p-value for these categories are indicated as a range because each category contains sub-functions that have their own p-value.

In addition, we studied the **molecular networks** that were assigned to both the “Mouse” and “Human” “high RPE gene expression” datasets. Functions annotated to these datasets on a network level were more or less comparable (~75% overlap). The annotated functions included developmental disorders, hereditary disorders, small molecule/drug metabolism and cellular movement and maintenance. For an overview of the 10 most important networks for the “Mouse high RPE gene expression” dataset and the “Human high RPE gene expression” dataset, and to see which networks overlap, see S3 Table and S4 Table. For illustrative purpose we included an example of such a network (S3 Fig). For additional support of our findings in Ingenuity we also included a functional enrichment pathway analysis (KEGG analysis) in Webgestalt [16]. This gives approximately the same results (S5 Table).

### Genes highly expressed in mouse RPE but hardly in human RPE

To investigate the largest gene expression and functional differences between the mouse and human RPE we subsequently compared the most extreme gene expression datasets of the two species, namely the very high (highest 10^th^ percentile, >90^th^P; high expression) and very low (lowest 10^th^ percentile <10^th^P; leaky expression) RPE expression datasets (GSE 66916).

Unexpectedly, the “Mouse high RPE gene expression” dataset (>90^th^ P, 2663 genes) and the “Human very low RPE gene expression”dataset (0-10^th^ P, 1770 genes) had 101 genes in common (S6 Table). Functional annotation of these genes yielded 31 **canonical pathways** in Ingenuity, whose activity or metabolic route may be differentially affected in mouse and human RPE. An overview is presented in Table 4.

**Table 4:** Overview of significant canonical pathways assigned by the Ingenuity knowledge database to the 101 genes that are the result of comparing the “Mouse high RPE gene expression” and the “Human very low RPE gene expression” datasets.

**Endocrine Signaling & Metabolic Function**
Ephrin Receptor Signaling
PEDF Signaling
Protein Kinase A Signaling
Gαq Signaling
FGF Signaling
Phospholipase C Signaling
NGF Signaling
GNRH Signaling
PXR/RXR Activation
Ephrin B Signaling
**Immunological Function**
iCOS-iCOSL Signaling in T Helper Cells
Role of NFAT in Regulation of the Immune Response
Dendritic Cell Maturation
B Cell Receptor Signaling
IL-8 Signaling
Thrombin Signaling
PKCθ Signaling in T Lymphocytes
CD28 Signaling in T Helper Cells
Role of Macrophages, Fibroblasts and Endothelial Cells in Rheumatoid Arthritis
GM-CSF Signaling
PI3K Signaling in B Lymphocytes
**Basic pathways of cellular (dys)function**
Prostate Cancer Signaling
Regulation of the Epithelial-Mesenchymal Transition Pathway
Wnt/Ca+ pathway
P2Y Purigenic Receptor Signaling Pathway
Estrogen-Dependent Breast Cancer Signaling
Colorectal Cancer Metastasis Signaling
**Epithelial junctions**
Tight Junction Signaling
Epithelial Adherens Junction Signaling
**Vesicle mediated transport**
Clathrin-mediated Endocytosis Signaling
**Oxidative stress**
Hypoxia Signaling in the Cardiovascular System

The core analysis of the 101 differentially expressed genes resulted in 7 **molecular networks**. The associated representative functions include developmental disorders, connective tissue disorders, ophthalmic disease, neurological disease, drug metabolism and cancer. These networks are presented in S7 Table. For illustrative purpose we included an example of one of these networks, see S4 Fig. For additional support of our findings in Ingenuity we also included a functional enrichment pathway analysis (KEGG analysis) in Webgestalt. This gives approximately the same results (S8 Table).

### Genes highly expressed in human RPE but hardly in mouse RPE

In order to identify additional differences between mouse and human RPE, we also compared the “Human high RPE gene expression” dataset (>90^th^ P, 2399 genes) and the “Mouse very low RPE gene expression”dataset (10^th^ P, 3374 genes). This analysis yielded 54 genes (S9 Table). We also functionally annotated this set of genes using the Ingenuity knowledge database. The significant **canonical** pathways assigned to this dataset included PXR/RXR activation, nicotine degradation and bupropion degradation. Ingenuity analysis yielded four networks. The functional annotations of these networks include drug metabolism, nucleic acid metabolism, small molecule biochemistry, cardiovascular disease and humoral immune response. The molecular pathways are presented in S10 Table. We also included an illustrative example of such a network, see S5 Fig.

Among the major **biological functions** and disease that came out of this analysis were hereditary hearing loss and Usher syndrome. Major differences in **molecular cellular functions** identified by Ingenuity were drug metabolism, nucleic acid metabolism, small molecule biochemistry and lipid metabolism. For additional support of our findings in Ingenuity we included a functional enrichment pathway analysis (KEGG analysis) in Webgestalt. This gives approximately the same results (see S11 Table).

## Discussion

In this study, we aimed to find similarities and differences between mouse and human RPE using RPE specific gene expression profiles and functional annotation on the same experimental platform. Our current data may be important for translational studies in age related macular degeneration, for creating and use of a representative AMD mouse model. Thus, we discuss here those aspects of our analyses of human and mouse RPE that are relevant for AMD.

### Similarities and differences between mouse and human RPE transcriptomes in relation to AMD

Apart from the obvious similarities, there are a number of well-known differences between human and mouse RPE and adjacent tissues. These include the absence of a macula in the mouse, the difference in rod and cone number and distribution, and a thinner Bruch’s membrane in the mouse. Mouse models are available for wet and dry AMD, mimicking several of the pathological features seen in AMD, but no model recreates all of the AMD characteristics [17–20].

We were interested in the potential usefulness of our entire comparative human and mouse gene expression dataset for the investigation of AMD (mouse models). Interestingly we did find similarities and differences in relation to a number of previously published (patho-) biological aspects related to AMD, namely oxidative stress, zinc homeostasis, presence of proteins of the complement system that are found in drusen, proteins in Bruch´s membrane, involvement in neovascularization and tight junctions. These differences and similarities are important to develop and use representative mouse models for AMD, and they may be partly responsible for (the observed) discrepancies between mouse model and human patients.

#### Age related macular degeneration: Oxidative Stress

The RPE suffers from chronic oxidative stress due to the exposure to light, relatively low oxygen levels, and daily phagocytosis and digestion of photoreceptor outer segments [21]. The mainstream hypothesis in AMD is that prolonged oxidative stress harms the vitality of the RPE and oxidatively modified drusen-bound fatty acids and proteins. These are subsequently recognized by the body as non-self, and invoke a chronic, complement mediated, immune response [22,23].

Oxidative stress in the RPE is, among others, mediated by the manganese superoxidase dismutase protein family, consisting of SOD1, SOD2 and SOD3; Respectively, these SODs exert their antioxidant effect in the cytosol, mitochondria and extracellular matrix [24,25]. We found similar expression of SOD1 (very high) and SOD3 (moderate) in human and mouse RPE. In contrast, we found that the SOD2 gene was highly expressed in the mouse RPE but only at low levels in the human RPE (Table 5). Reactive Oxygen species (ROS)-associated mitochondrial DNA damage was previously correlated with the progression of AMD [26–28]. But association studies between genetic variants in the SOD2 gene and AMD pathogenesis yielded conflicting results [27,29].

**Table 5:** SOD1, SOD2, SOD3 gene expression in human and mouse RPE.

	Mouse		Human	
isoenzyme	Reporter	Percentile	Reporter	Percentile
SOD1	NM_011434	High	NM_000454	High
SOD2	NM_013671	High	NM_000636, BM994509, AL050388	Low
SOD3	NM_011435	Intermediate	NM_003102	Intermediate

Sod1 and Sod3 are highly and moderately expressed respectively, in both species. Sod2 gene expression has a low expression in human RPE. In contrast it has a high expression in mouse RPE.

For both SOD1 and SOD2 mouse models were developed. *Sod1-/-* mice and *Sod2-/-* mice both show a thickened Bruch’s membrane, photoreceptor atrophy and reduced electroretinographic response [30]. *Sod2-/-* mice lacked drusen like deposits but have RPE atrophy [31,32]. In the *Sod1-/-* mice, 10% of the older animals showed choroidal neovascularization and 86% showed drusen-like deposits that contained several markers of drusen [33].

The previous studies on SOD family members and the different expression we find between mouse and human, indicate that all three SOD family members may be critically involved in the local defense against oxidative stress, although the mitochondrial SOD2 may play a more important role in the mouse RPE than in the human RPE.

In addition to SODs, zinc has also been implicated in mediating oxidative stress. The retina and especially drusen contain high amounts of zinc [34]. There is an age related decrease in systemic and cellular zinc levels in human RPE cells, that correlates with several age related pathologies like AMD [35]. In 1988 the first clinical trial favoring zinc supplementation in AMD was published [36]. Since that time, multiple studies suggested that zinc reduces the oxidative burden on the retina although the underlying molecular mechanism(s) is unknown [7,37–39]. Zinc ions reach the retina by specific transporters. We determined which zinc transporters are highly expressed in mouse and human RPE. In the highest 10^th^ percentile of the mouse RPE transcriptome we found expression of *Slc39a1*, *Slc39a4*, *Slc39a7* and *Slc39a12*. In the highest 10^th^ percentile of the human RPE transcriptome we observed expression of *SLC39A8*, *SLC39A12* and *SLC39A13* (Table 6). Our data are largely in agreement with those of Leung and coworkers [40], who determined the expression of a large number of zinc transporters in cultured human RPE cells.

Interestingly, we found that *Slc39a4* was highly expressed in mouse RPE, but not in the human RPE. Indeed, Dufner-Beattie et al demonstrated the importance of this transporter in a *Slc39a4* knockout mouse, which develops severe abnormalities of the nervous system, such as anophthalmia, exencephaly and hydrocephalus [41]. Our finding (in older human donor eyes) may be explained by an age-related effect, since Leung et al [40] found that *Slc39a2* and *Slc39a4* expression and corresponding zinc uptake are reduced in RPE from older individuals. We determined the *Slc39a4* expression in RPE of five-month-old mice.

**Table 6:** Overview of zinc transporters that are highly expressed in human and mouse RPE.

Mouse		Human	
Zinc transporter	reporter	Zinc transporter	reporter
*Slc39a1*	NM_013901	*SLC39A8*	NM_022154
*Slc39a4*	NM_028064	*SLC39A12*	NM_152725
*Slc39a7*	NM_008202	*SLC39A13*	NM_152264
*Slc39a12*	NM_001012305		

#### Age-related macular degeneration: drusen and complement system

**Fig 5:**
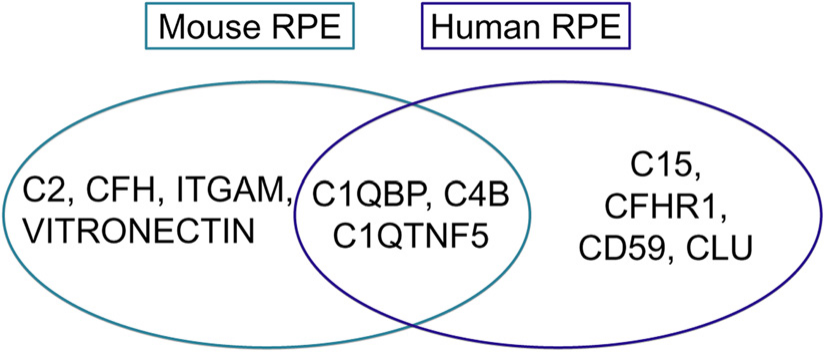
Overview of highly expressed complement factors in the human and mouse RPE. Complement factors in the overlay of the circles are highly expressed in RPE of both species.

Chronic inflammatory and immune mediated events at the level of the Bruch’s membrane and drusen play critical roles in AMD pathogenesis [42]. Initially, complement system related factors were immune-localized to drusen, a hallmark of AMD. Subsequently, genetic studies showed an association between polymorphisms of several complement pathway genes, such as *CFH*, *CFB*, *C3*, *CFHRs*, and AMD [2]. In our dataset we found high expression of several complement factors in the human and mouse RPE (Fig 5), which may be of interest for studies of the complement system and AMD pathogenesis in a mouse model.

Interestingly, C1QTNF5 is highly expressed in the both mouse and human RPE. Mutations in C1QTNF5 have been associated with late-onset retinal degeneration. A *C1qtnf5 S163R* knock-in mouse model developed by Chavali et al showed many pathological features of AMD, such as RPE abnormalities, photoreceptor loss, retinal vascular leakage [43]. Contrary to this, Shu et al. developed a *C1qtnf5 Ser163Arg* knock-in mouse model that lacked any phenotypic abnormality [44]. The reason for this discrepancy is currently not clear.

We observed that the complement factor H related 1 gene (CFHR1) is highly expressed in human RPE, but not in mouse RPE. Our data corroborate, in part, the data of Luo et al (*2011*) who found absence of *Cfhr1* expression in mouse retina, RPE and choroid [45]. The regulation of the complement system (in AMD) is extremely complex, multiple regulators and feedback loops exist and the detailed mechanisms underlying the complement regulation at the RPE interface and macular area is not known. Nonetheless, several studies suggested that CFHR1, together with CFHR3, plays a central role in complement regulation of AMD. Several studies suggest that the absence of CFHR1 and/or its family member CFHR3 are highly protective against AMD in humans [46–48].

#### Age-related macular degeneration: Bruch’s membrane and neovascularization

Our data reveal differences in mouse and human RPE gene expression related to two other essential aspects of age-related macular degeneration: The build-up and turnover of Bruch’s membrane and neovascularization. Bruch’s membrane is a sheet of extracellular matrix that lies in between the RPE and the choroid. The extracellular matrix components that form the BM are made by RPE and CH. The BM has a major clinical significance because of its critical role in the pathogenesis of AMD [2].

We found that *Timp2* and *Col3a*, genes involved in extracellular matrix formation or turnover, are highly expressed in the mouse RPE, but not in human RPE. Vice versa, *COL16A1* is highly expressed in the human RPE, but not in mouse RPE.

We also found large inter-specifics gene expression differences annotated with the term “Angiogenesis”. “Angiogenesis” refers to the process whereby new blood vessels are formed. In the context of our RPE/AMD analysis, choroidal blood vessels usually penetrate the BM and from new (leaky) vessels underneath the RPE. Our data specifically suggest expression and functional differences for the angiogenic factors *Fgf23* and *Fgfr1* (highly expressed in mouse RPE), as well as the prostaglandin synthase *PTGES* and *HS6ST1* (highly expressed in human RPE). In summary, our results suggest specific differences between mouse and man in terms of BM buildup or turnover, as well as related to neovascularization.

#### Age-related macular degeneration: tight junctions of the outer blood-retina barrier

**Fig 6:**
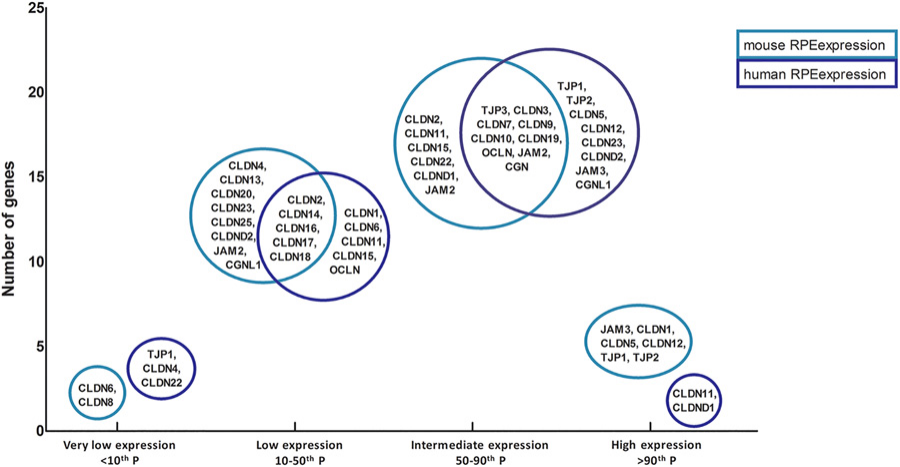
This diagram depicts the tight junction gene expression of mouse and human RPE, divided in four categories: high expression (>90^th^ percentile), moderate (50-90^th^ percentile), low (10-50^th^ percentile) and very low (<10^th^ percentile). On the x-axis the four categories are displayed and on the y-axis the amount of genes found in a category is depicted. Light blue circles contain genes expressed in mouse RPE, Dark blue circles genes expressed in human RPE. Genes inside the overlapping parts of the circles are expressed in the RPE in both species in that category.

The RPE constitutes the outer blood-retina barrier (oBRB). The tight junctions between neighboring RPE cells bind the monolayer and separates the outer layer of the neural retina from the choriocapillaris [49]. The RPE maintains the integrity of the oBRB through the tight junctions, which is important for control of fluid leakage, solute transport and immune reactions. oBRB supports the functional homeostasis of the retina. Disruptions of RPE cell junction and barrier integrity are associated with AMD [50,51]. We compared the tight junction gene expression of mouse and human RPE by investigating the distribution of these genes in four categories: high expression (>90^th^ percentile), moderate (50-90^th^ percentile), low (10-50^th^ percentile) and very low (<10^th^ percentile) (Fig 6).

Overall, we find limited overlap of tight junction gene expression between mouse and human RPE. Our data suggest that the composition of the outer blood retina barrier differs between mouse and human. More investigation is necessary to determine the possible physiological or pathobiological effect of these differences.

## Conclusion

In summary, in this study we determined 64 signature genes for mouse RPE, 171 signature genes for human RPE. We also deduced 22 mouse-human interspecies signature genes. We next analyzed the general mouse and human RPE gene expression profiles, and we found that (patho-) biological functions and canonical pathways assigned to the RPE of both species were highly similar. Nonetheless, more detailed studies, including analysis of specific molecular networks as well as extreme gene expression differences between mouse and human (expression of 155 genes), suggests substantial biological differences.

These similarities and differences may be important for the development of new therapeutic strategies and translational studies in age-related macular degeneration.

## Methods

### Strengths and limitations of the study design

The technical and methodological strengths and limitations of this approach have been extensively discussed elsewhere [12,13]. Our lab has more than 10 years’ experience in cellular microarray studies. In short, the strengths of this study include the use of selected healthy and freshly frozen samples with short post-mortem times. Sample preparation is characterized by minimal technical handling (such as mechanical or enzymatic dissociation, scraping, heating etc). In this way, the native “*in vivo*” gene expression profile is preserved. Next, we use laser dissection microscopy (LDM) of cryosections of the relevant cell-type. The use of the LDM ensures highly specific and homogeneous cell-type collection. After RNA isolation, we check RNA integrity and the quantity using the Agilent Bio-analyzer and Nanodrop during the procedure multiple times. Samples are labeled with Cy3 and Cy5 from the 3’-prime end to minimize effects from possible RNA degradation. RNA/samples that do not meet our quality criteria at any point in the procedure are discarded of. We use a common reference design, which also serves as an internal technical control, and a large-scale 44k microarray.

There are also a number of limitations of our studies. Given the lengthy procedure of sample selection, procedure and extensive quality controls, we usually include a limited number of “the very best samples” in our final microarray analysis. The consequence is that we only can detect consistent similarities and differences (and not all, strongly variable or transient ones) in gene expression between samples.

Another limitation is that some degree of cellular contamination of adjacent cell layers in samples is unavoidable, even when we use meticulous laser dissection microscopy in the nicely structurally stacked retina. To overcome this problem we included the gene expression of the adjacent layers in our analysis: the so-called “double selection procedure” (Fig 2) [12].

There are two limitations which are specific to this mouse-human study: The first is that there may be an oligo design difference for the comparative orthologous human and mouse genes on the Agilent whole Mouse and whole Human microarray. While, frequently, multiple different oligo’s for a single gene and reference genes may be present on the micro-array, this may hamper direct comparison between mouse and human gene expression data. To overcome this problem, we ranked the gene expression data of each sample/species according to percentiles, and divided it into four expression groups: high, moderate, low and very low expression [13,52]. By comparing the most extreme datasets, the high expressed genes with the very low expressed genes, between the two species; we could identify physiological relevant differences between the mouse and human RPE gene expressions, since these major differences could not be caused by different affinities alone. For further confirmation, we identified the genes that we described in our paper also by sqRT-PCR.

Finally, the mouse and human tissue used in this study had different post-mortem times: The mouse eyes were enucleated and embedded immediately after death, while for the donor eyes the post-mortem delay was between 16 and 22 hours. On the other hand, it has been shown that this has minimal effect on the RNA integrity of brain tissue. Up to 30 hours postmortem delay did not affect the mRNA [53]. During our experiments, we thoroughly checked the RNA integrity using BioAnalyzer, multiple times. In addition, since we designed labeling primers on the 3 prime end of the genes, potential starting degradation (first occurring at the 5 prime end) did not affect our gene expression results. A full description of the methodological (dis) advantages is beyond the scope of this paper. However, our approach enables us to determine highly specific RPE gene expression with a very limited amount of contamination, which is also corrected for in the analysis.

### Mouse eyes, tissue processing and cell sampling

The study on mouse material was carried out in strict accordance with the recommendation in the Guide for the Care and Use of Laboratory Animals under the Dutch law, which is in accordance with the international declaration of Helsinki. The protocol was approved by the Committee on Ethical of Animal Experiments of the Netherlands Institute for Neuroscience (NIN), Royal Dutch Academy for Science (KNAW), the Netherlands (DEC protocol NIN 09.45). Mouse choroid, RPE and photoreceptors were obtained from eyes of healthy 5 months (-/+2 weeks) old C57BL/6 mice (J strain). We confirmed by sqRT-PCR that the mice of this sub strain (C57BL/6JOlaHsd) did not carry the rd8 mutation in the *Crb1* gene that has been found in C57BL/6N strains [54]. For each tissue we used 3 mouse eyes (and 3–6 selected human donor eyes). Mice were raised in a room with a temperature around 21°C, on a 12:12-h light-dark cycle, and fed with standard pellet laboratory chow and water ad libitum. By the age of 5 months (-/+2 weeks) they were anesthetized with CO_2_/O_2_ and killed by cervical dislocation. The eyes were enucleated, embedded in OCT and snap frozen in liquid nitrogen. Until further use the eyes were stored in an -80°C freezer. We selectively cut out the CH, RPE and PR with a laser dissection microscope (PALM Carl Zeiss, MicroImaging GmbH, Munich, Germany). For this the eyes were cut in 20uM cryosections for the photoreceptors and 12uM for the RPE and the choroid. For every sample one whole eye was used. We used Cresyl Violet staining to identify photoreceptor cells. Before the dissection of the choroid the RPE was removed to prevent as much contamination as possible. After processing the tissue and running the microarray, we determined the (low) variability of the samples using a multidimensional scaling plot (S6 Fig).

### RNA isolation and amplification

RNA isolation, amplification and labelling procedures were carried out essentially as described elsewhere [14]. Quality of tRNA was checked with a Bioanalyzer assay (RNA 6000 Pico Kit, Agilent Technologies, Amstelveen, The Netherlands). RNA integrity numbers of tRNA of mouse CH ranged from 4.9 to 6.9, of the mouse RPE ranged from 4.9 to 7.2 and of mouse PR ranged from 5.5 to 7.6. In our microarray study we used a common reference design. The common reference was prepared from mouse RPE/choroid that was isolated, amplified using the same methodology as our experimental samples, and labelled with Cy3 (Cy3 mono-reactive dye pack, GE Healthcare UK, Little Chalfont, Buckinghamshire, UK).

See Janssen et al. [14] for a more detailed description of the laser dissection procedures, RNA processing and microarray procedures.

### Microarray data analysis

The microarray files were analysed and processed using Agilent Feature Extraction Software (Agilent Technologies, version 9.5.3.1). We included examples of our strict quality control assessment of the hybridizations in the supplementary file S7 Fig and S8 Fig. Data were imported into R (version 2.14.0 for Windows, R Development Core Team, 2009) using LIMMA in the Bioconductor package. We studied the differences between the RPE and the photoreceptors making a statistical comparison using LIMMA for determining significantly changed genes (R package LIMMA, including Bayesian statistics). We did the same for RPE and the choroid:

Using a common reference design, in LIMMA we first estimated the difference between the sample (either CH / RPE / PR) and the common reference (hybridized against each other on a two channel array). Next, the differences between both RPE and CH or between RPE and PR were estimated. Subsequently, LIMMA fitted a linear model to the expression data for each gene. LIMMA uses empirical Bayes statistics to moderate the standard error of the estimated log-fold changes which results in more stable inference and improved power [55]. A fully detailed description of the script that was used in LIMMA is available upon request.

We selected the genes that had a positive fold change, meaning that they are higher expressed in the RPE than in either the photoreceptors or the choroid, of more than 2.5. Cut-off value for statistical significant difference was an adjusted p-value of less than 0.01 after Benjamini-Hochberg correction for multiple testing. Two data subsets were created that either contained all genes that show a significant higher expression in the RPE compared to the photoreceptors (RPE>PR), or a significant higher expression in the RPE compared to the choroid (RPE>CH). The volcano plots that visualize the symmetrical spread of the differentially expressed genes are included in S9 Fig. Next we compared these two subsets to determine the genes that are present in both lists using a comparison analysis in IPA (Ingenuity Systems). These represent RPE specifically expressed genes. To visualize these significant differences in gene expression levels we included a figure depicting the mean and (low) standard deviations (S10 Fig) and we included a figure of the mean and (the low) standard deviations of the genes mentioned in the discussions section (S11 Fig).

### Data analysis of two microarray studies on the human RPE transcriptome

To detect possible contamination of the choroid and the photoreceptors in the list of Strunnikova et al we used the expression data of the human choroid and the human photoreceptors as determined within our group [12]. We assumed that the main source of contamination of RPE sample(s) using this methodology comes primarily from the set of highest expressed genes in either the PR or CH. Consequently, we determined the highest 10^th^ percentile of the average gene expression for both the photoreceptors and the choroid in Microsoft Excel. We ran a comparison analysis in IPA (Ingenuity Systems) to subtract the genes in the highest 10^th^ percentile of photoreceptors and choroid from the 154 genes determined as RPE specific by Strunnikova et al [15]. We merged the two human RPE specific gene expression lists in Ingenuity using a comparison analysis.

### Interspecies RPE signature genes

We compared the human RPE signature gene expression list, that we determined using the *Booij et al* list and the corrected *Strunnikova et al* list (Fig 3), with our new mouse RPE signature gene expression. We ran a comparison analysis in Ingenuity, which uses the Entrez Gene identifier, to investigate which genes are found in both datasets and thus are interspecies specific.

### Confirmation of gene expression data by sqRT-PCR

We confirmed our microarray data with sqRT-PCR. For a detailed description of the sqRT-PCR, see Janssen et al. 2013 [13]. In short, sqRT-PCR was carried out using intron-spanning primers on cDNA from laser dissection microscopy derived samples, using three biological replicates. To minimize effects of RNA degradation artefacts, we generated primers near the 3’end of the gene. We quantified the gene expression in ImageJ and normalized expression by comparing it to the measured expression of housekeeping gene *Bact*.

Previously, we confirmed the human gene microarrays [12]. In the current study study, we selected a total of 27 genes to confirm our mouse microarray data. First, we selected randomly 11 highly expressed genes from our “Mouse RPE signature genes” dataset (*Bmp4*, *Rlbp1*, *Rgr*, *Krt18*, *Sgk3*, *Man1a*, *F3*, *Sulf1*, *Thsr*, *Col4a4*, *SLc16a8*). For 9 out of the 11 “Mouse RPE signature genes” we found the highest expression levels in the mouse RPE and the lowest in the CH and PR (Fig 1). Only *Thsr* and *Slc16a8* showed highest expression in CH.

We next selected 8 well-established RPE specifically expressed genes (*Mertk*, *Rrh*, *Tyr*, *Rpe65*, *Rdh5*, *Lrat*, *Tjp1 and Trpm3*). For 7 out of 8 of the well-established RPE specific genes, we found highest expression in the mouse RPE and lower in the CH and PR (S1 Fig). Only *Tyr* showed highest expression in CH in our RT-PCR.

We also included sqRT-PCR for genes that were mentioned in the Discussion section to further technically validate our microarray. We selected 8 genes, 6 genes that are highly expressed (found in the highest 10^th^ percentile of the mouse RPE microarray; *Sod1*, *Sod2*, *Slc39a4*, *Timp2*, *Col3a*, *Cldn1*) and 2 genes that are low expressed (found in the lowest 10^th^ percentile of the mouse RPE microarray; *Cldn8*, *Hs6st1*). We compared the expression levels of these genes with the expression level of *Bact*. For all genes we found the expected confirmatory result (S2 Fig). Overall, in this study, we confirmed the expression levels for 24 out of 27 genes (89%), which is in line with the cumulative RT-PCR confirmation rate (87%) of all previous microarray studies (using similar tissue and methodology).

## Supporting Information

S1 Fig Confirmation of well-established RPE genes by sqRT-PCR. *Bact* was used to normalize gene expression in mouse CH, RPE and PR. The light blue bars indicate the CH, the blue bars indicate RPE and the dark blue bars indicate PR. Similar to the microarray data the expression level is highest in the RPE and lowest in the PR.
(TIF)

S2 Fig Confirmation of microarray results mentioned in the discussion by sqRT-PCR. Dark blue bar indicate *Bact* expression level, the blue bars show the expression level of genes that were highly expressed on the microarray en light blue bars indicate expression of genes that showed very low expression on the microarray.
(TIF)

S3 Fig Example of a molecular network generated by Ingenuity Pathway Analysis from the “Mouse high RPE gene expression” dataset. (TIF)

S4 Fig Example of a molecular network assigned by Ingenuity to the 101 genes that are the result of comparing “Mouse high RPE gene expression” and the “Human low RPE gene expression” dataset. (TIF)

S5 Fig Example of a molecular network assigned by Ingenuity to the 54 genes that are the result of comparing “Human high RPE gene expression” and the “Mouse low RPE gene expression” dataset. (TIF)

S6 Fig Cluster analysis using multidimensional scaling plot. We analyzed the normalized expression intensities of the nine samples that were used in this study, with a multidimensional scaling plot. It shows that the samples within a group are highly similar.
(TIF)

S7 Fig Feature extraction quality control, background correction. Plot of the log of the red background-corrected signal versus the log of the green background-corrected signal for non-control inlier features for all nine samples after hybridization. The linearity of the plots indicate the appropriateness of background method choices.
(TIF)

S8 Fig Feature extraction quality control, spatial distribution. The spatial distribution of up- and down-regulated features is evenly spread throughout the array for all nine samples.
(TIF)

S9 Fig Volcano plots of differential expression RPE-CH and RPE-PR. Shown is the spread of the differentially expressed genes derived from the t-tests. The positive (higher expression in RPE) and negative (higher expression in the CH or in the PR) fold changes are approximately symmetrically divided.
(TIF)

S10 Fig Expression intensities of the mouse RPE signature genes. Shown are the means and standard deviations in the mouse CH, RPE and PR.
(TIF)

S11 Fig Expression intensities of the genes mentioned in the discussions section. Shown are the means and the standard deviation in the mouse RPE. The upper line indicates the cut-off value for the highest 10^th^ percentile, the middle line the cut-off value for the 50^th^ percentile and the lowest line the cut-off value for the lowest 10^th^ percentile.
(TIF)

S1 Table Genes expressed high in CH vs RPE and PR vs RPE. (XLSX)

S2 Table 171 genes Human RPE signature genes. (XLS)

S3 Table Molecular networks of the Mouse high RPE gene expression dataset. (XLS)

S4 Table Molecular networks of the Human high RPE gene expression dataset. (XLS)

S5 Table Molecular networks of the Mouse and Human high RPE gene expression dataset in Webgestalt. (XLSX)

S6 Table 101 genes highly expressed in mouse RPE but hardly in human RPE. (XLS)

S7 Table Molecular networks of the 101 genes highly expressed in mouse RPE but hardly in human RPE. (XLS)

S8 Table Molecular networks of the 101 genes highly expressed in mouse RPE but hardly in human RPE in Webgestalt. (XLSX)

S9 Table 54 genes highly expressed in human RPE but hardly in mouse RPE. (XLS)

S10 Table Molecular networks of the 54 genes highly expressed in human RPE but hardly in mouse RPE. (XLS)

S11 Table Molecular networks of the 54 genes highly expressed in human RPE but hardly in mouse RPE in Webgestalt. (XLSX)
