# Supplementary figures and images for: Comparison of Mouse and Human Retinal Pigment Epithelium Gene Expression Profiles: Potential Implications for Age-Related Macular Degeneration
Source: PLoS One. 2015 Oct 30;10(10):e0141597. doi: 10.1371/journal.pone.0141597 (PMC4627757; doi:10.1371/journal.pone.0141597)

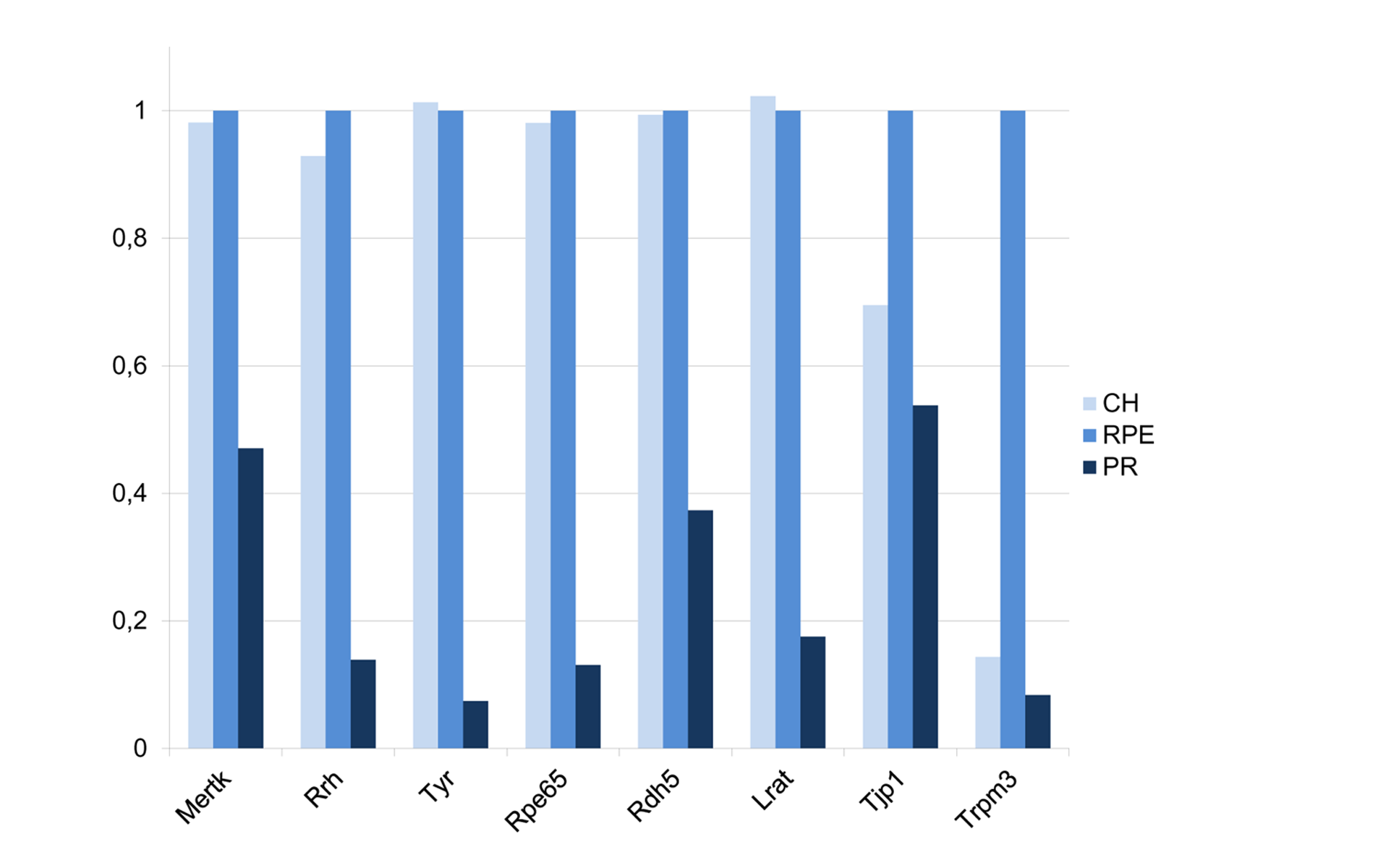

Supplement: S1 Fig — Bact was used to normalize gene expression in mouse CH, RPE and PR. The light blue bars indicate the CH, the blue bars indicate RPE and the dark blue bars indicate PR. Similar to the microarray data the expression level is highest in the RPE and lowest in the PR. (TIF) [file pone.0141597.s001.tif]

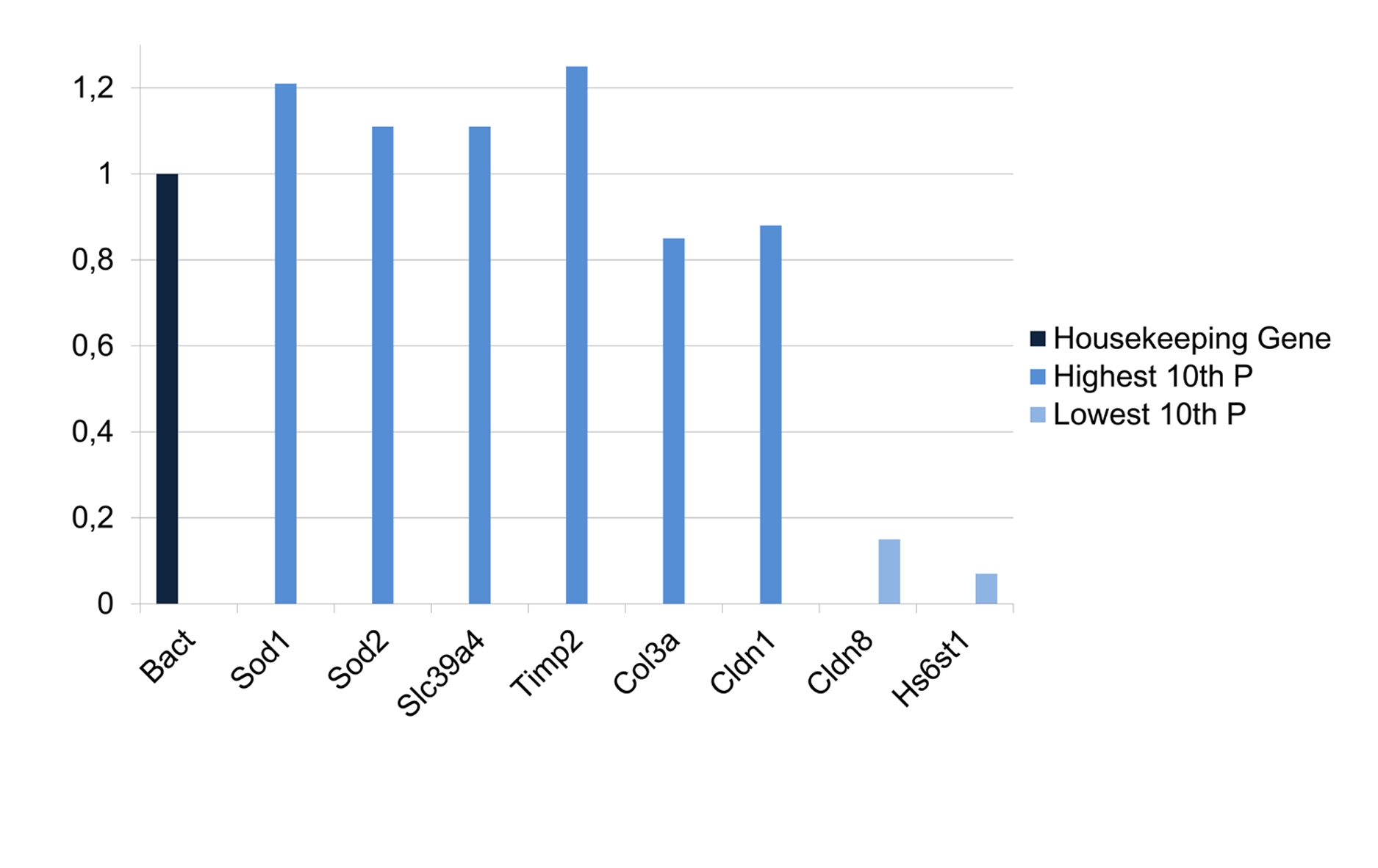

Supplement: S2 Fig — Dark blue bar indicate Bact expression level, the blue bars show the expression level of genes that were highly expressed on the microarray en light blue bars indicate expression of genes that showed very low expression on the microarray. (TIF) [file pone.0141597.s002.tif]

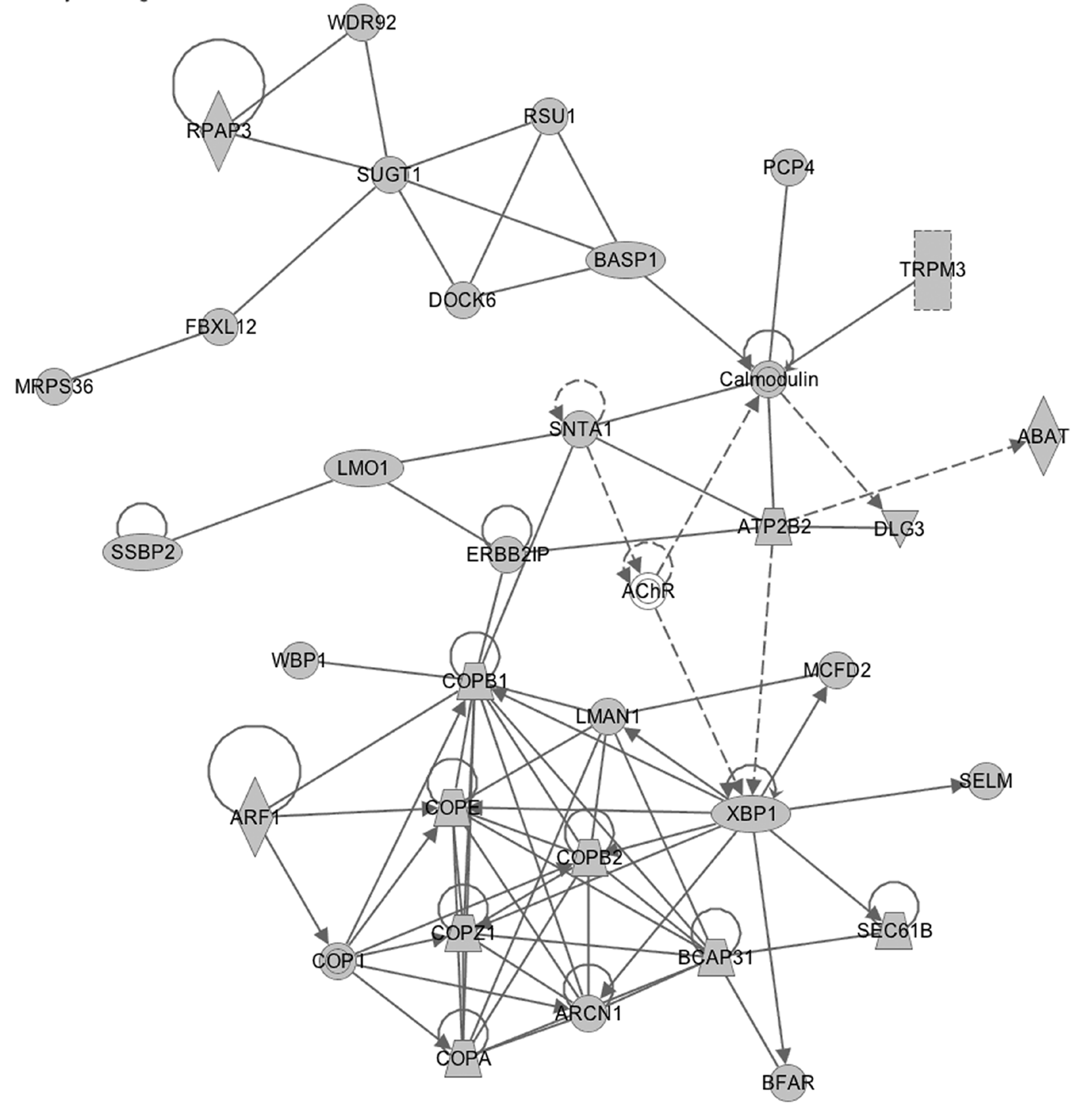

Supplement: S3 Fig — (TIF) [file pone.0141597.s003.tif]

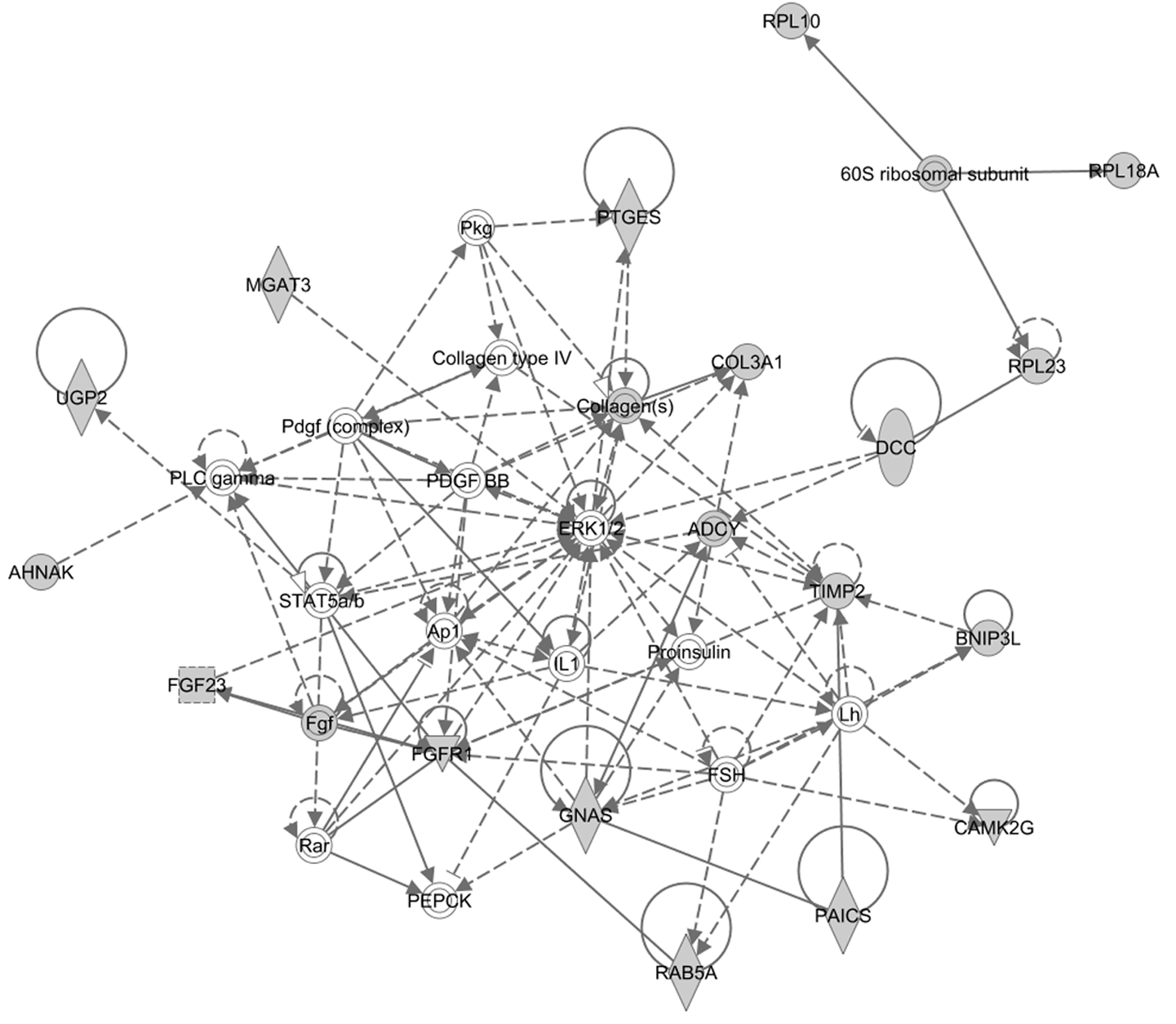

Supplement: S4 Fig — (TIF) [file pone.0141597.s004.tif]

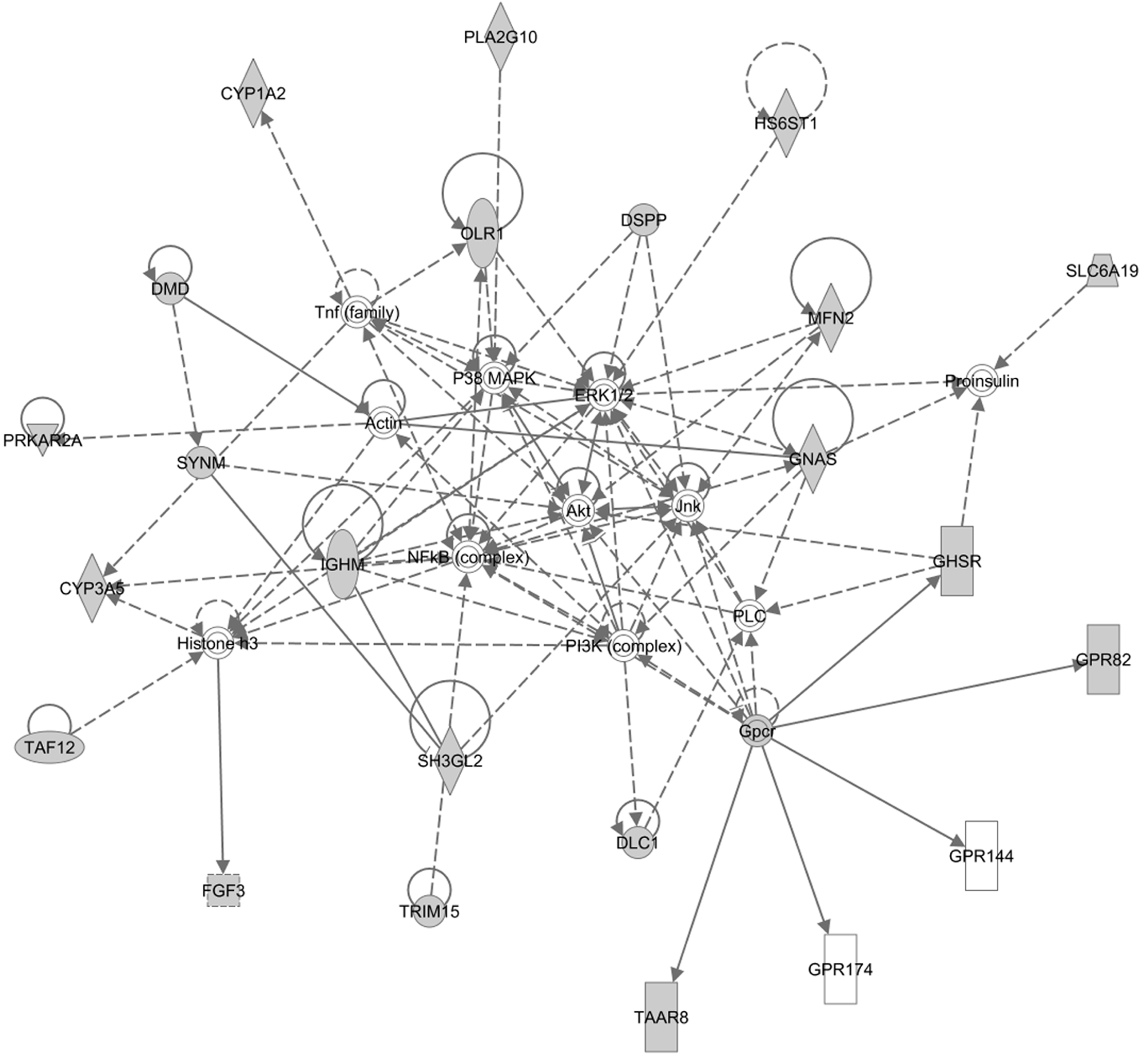

Supplement: S5 Fig — (TIF) [file pone.0141597.s005.tif]

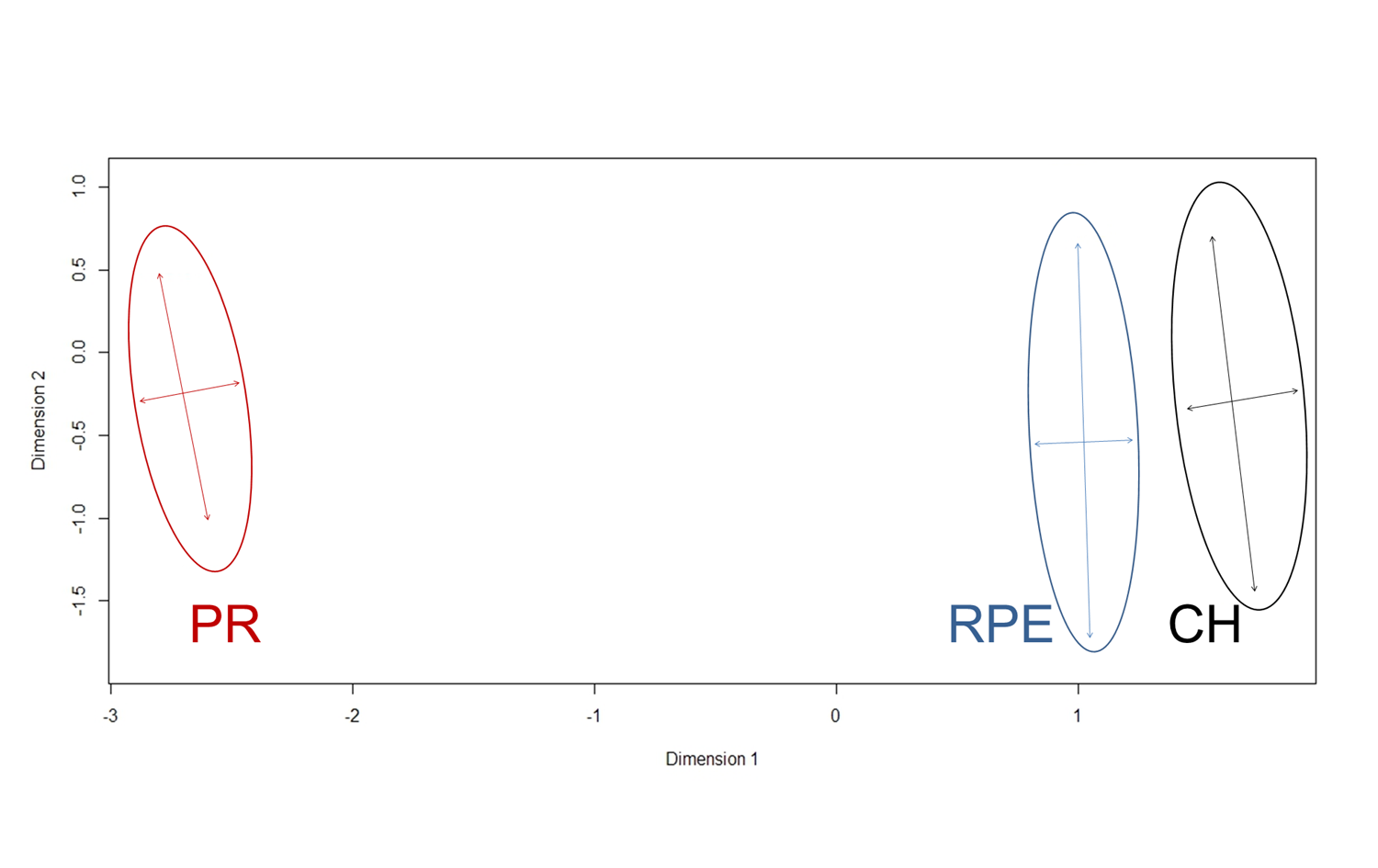

Supplement: S6 Fig — We analyzed the normalized expression intensities of the nine samples that were used in this study, with a multidimensional scaling plot. It shows that the samples within a group are highly similar. (TIF) [file pone.0141597.s006.tif]

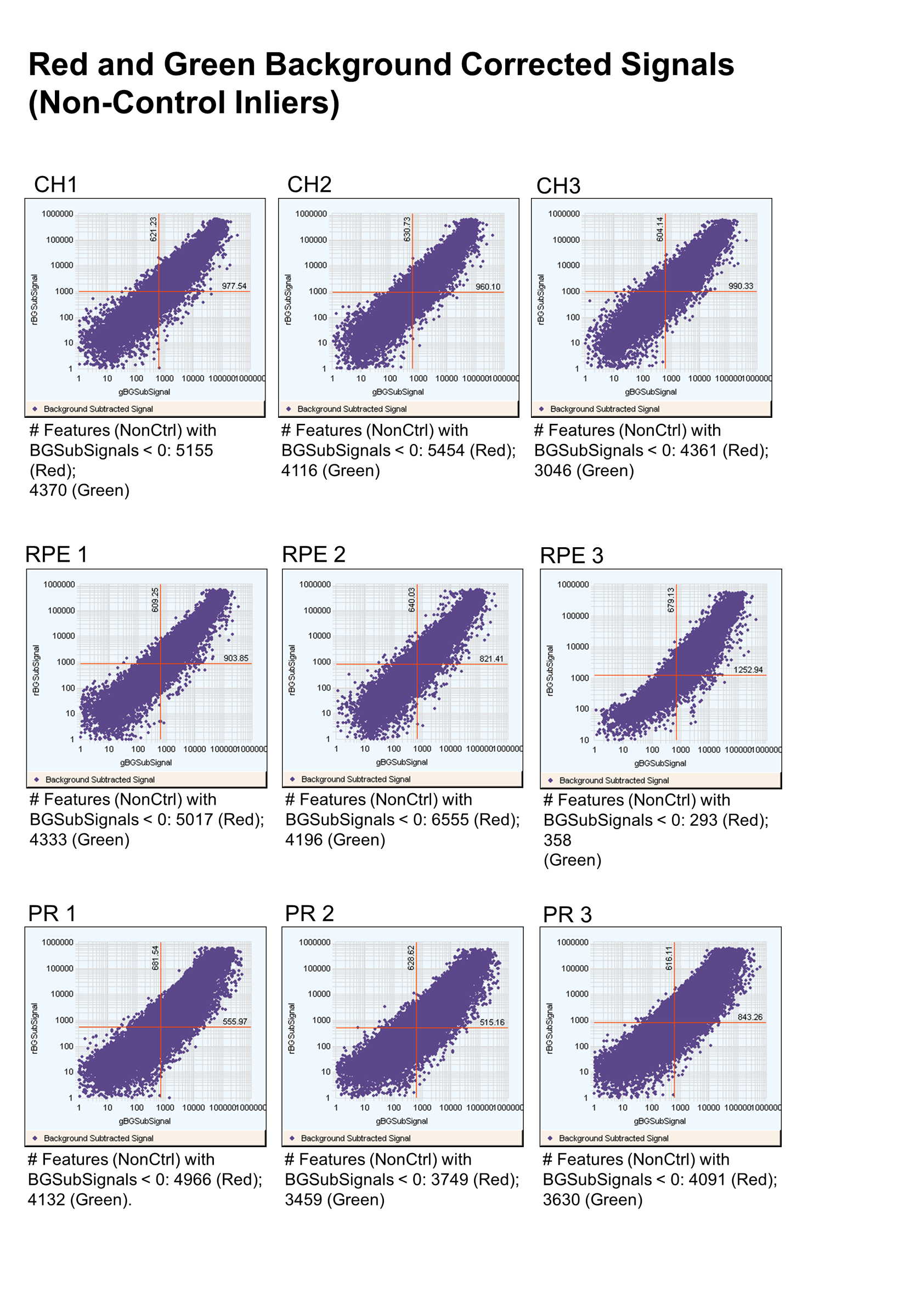

Supplement: S7 Fig — Plot of the log of the red background-corrected signal versus the log of the green background-corrected signal for non-control inlier features for all nine samples after hybridization. The linearity of the plots indicate the appropriateness of background method choices. (TIF) [file pone.0141597.s007.tif]

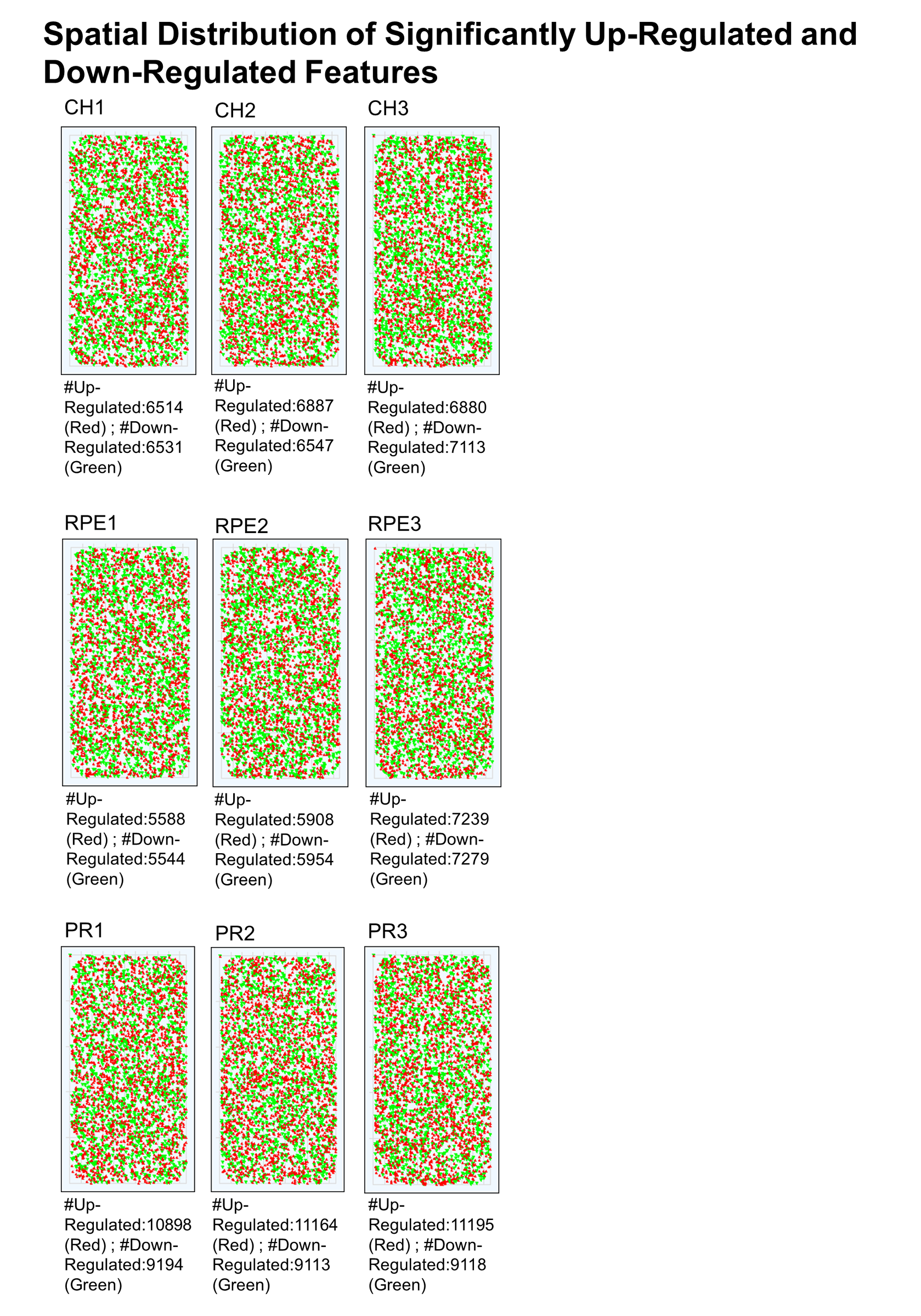

Supplement: S8 Fig — The spatial distribution of up- and down-regulated features is evenly spread throughout the array for all nine samples. (TIF) [file pone.0141597.s008.tif]

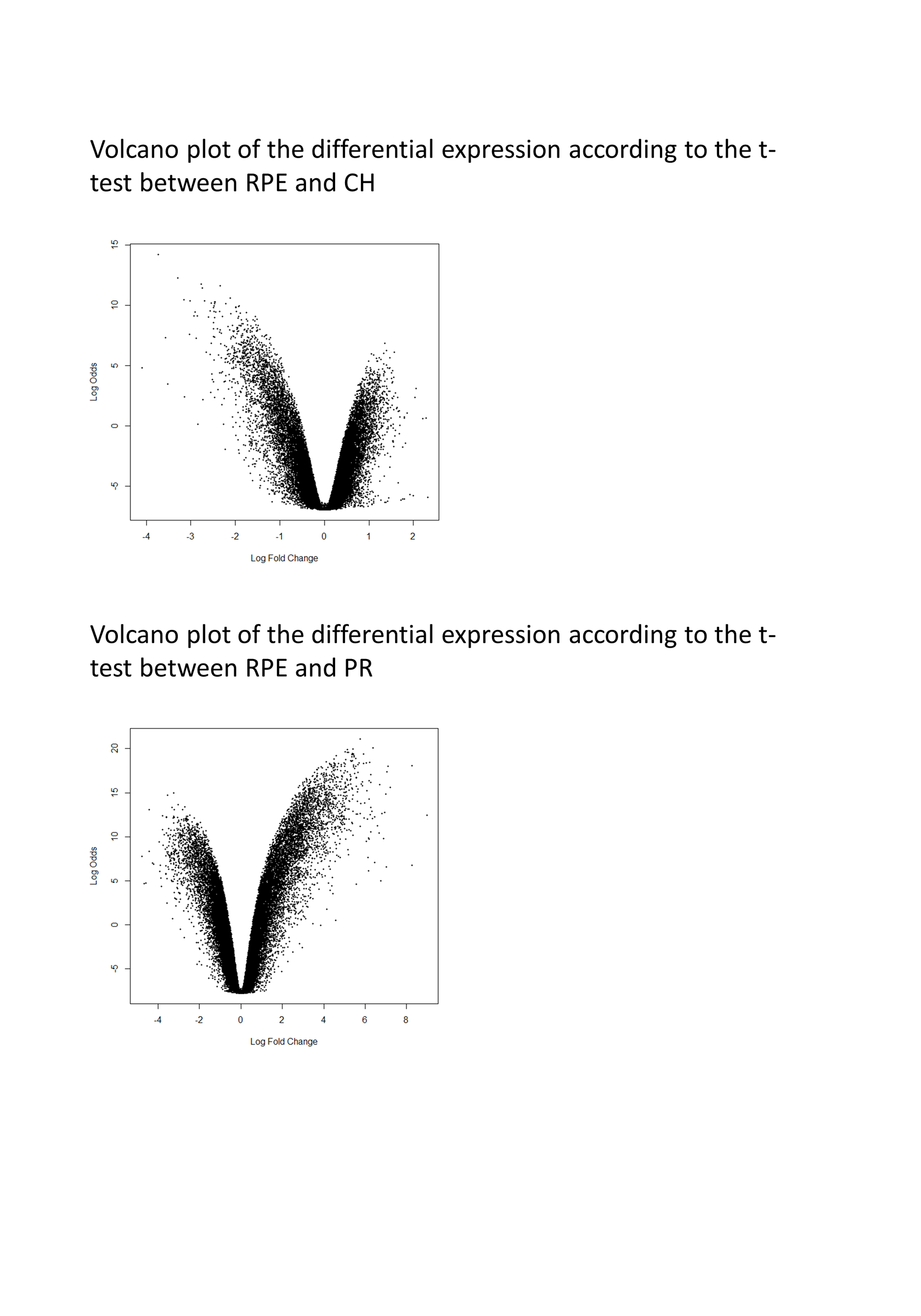

Supplement: S9 Fig — Shown is the spread of the differentially expressed genes derived from the t-tests. The positive (higher expression in RPE) and negative (higher expression in the CH or in the PR) fold changes are approximately symmetrically divided. (TIF) [file pone.0141597.s009.tif]

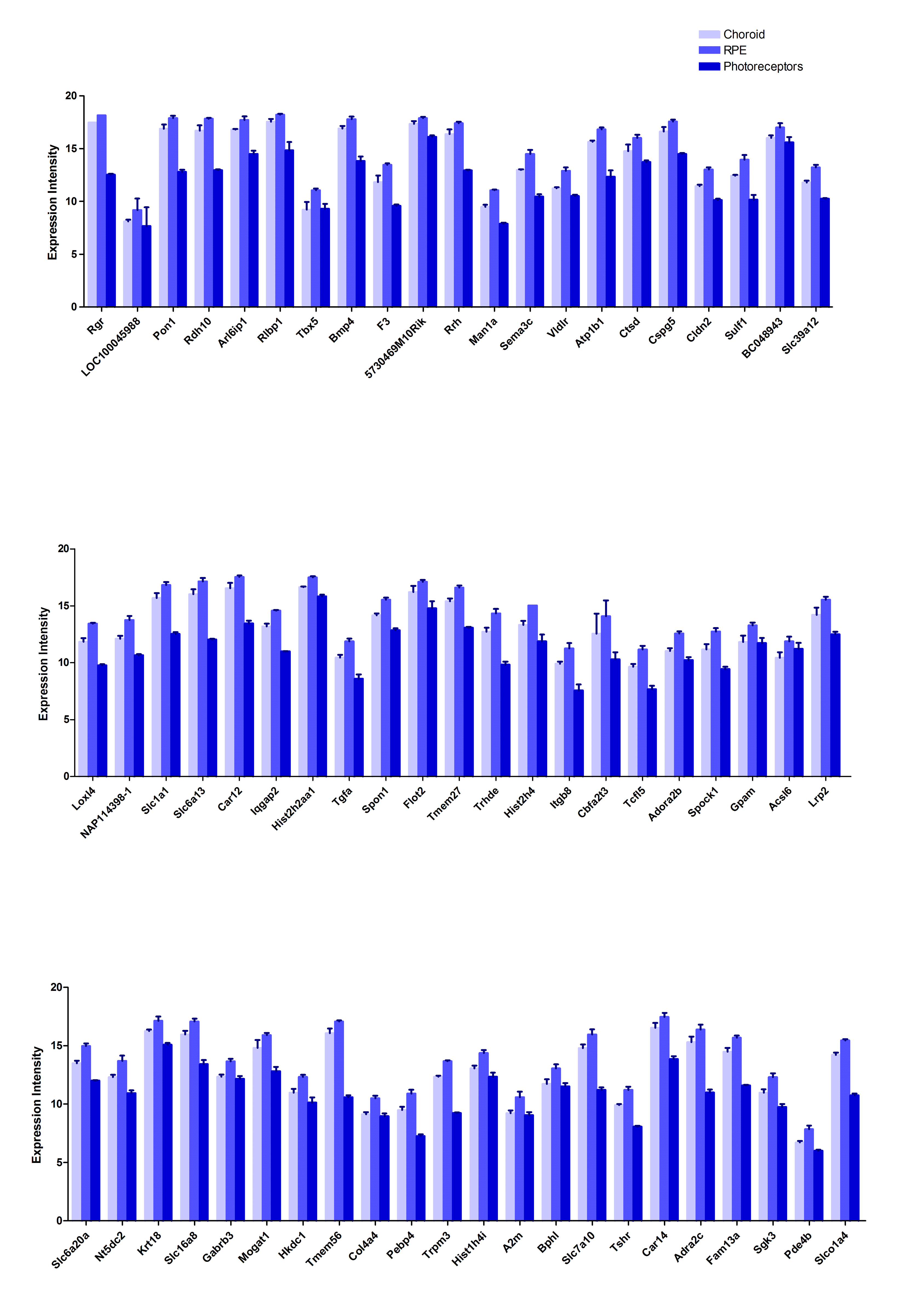

Supplement: S10 Fig — Shown are the means and standard deviations in the mouse CH, RPE and PR. (TIF) [file pone.0141597.s010.tif]

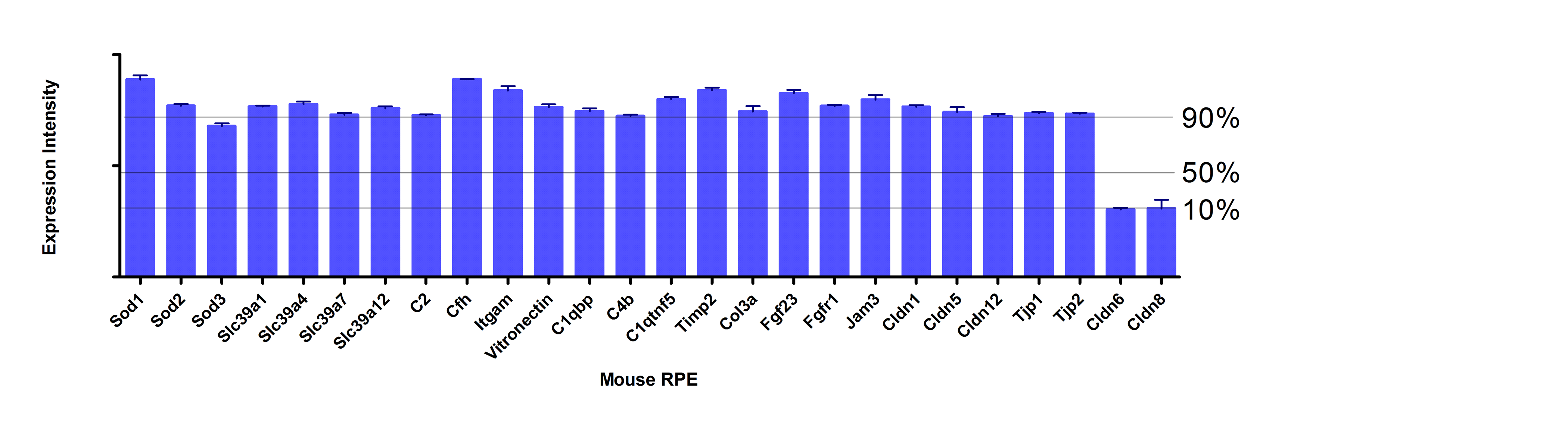

Supplement: S11 Fig — Shown are the means and the standard deviation in the mouse RPE. The upper line indicates the cut-off value for the highest 10th percentile, the middle line the cut-off value for the 50th percentile and the lowest line the cut-off value for the lowest 10th percentile. (TIF) [file pone.0141597.s011.tif]
